# Sentiment Classification of News Text Data Using Intelligent Model

**DOI:** 10.3389/fpsyg.2021.758967

**Published:** 2021-09-28

**Authors:** Shitao Zhang

**Affiliations:** School of Network Communication, Zhejiang Yuexiu University, Shaoxing, China

**Keywords:** transfer learning, text sentiment, cross-domain, intelligent model, sentiment classification

## Abstract

Text sentiment classification is a fundamental sub-area in natural language processing. The sentiment classification algorithm is highly domain-dependent. For example, the phrase “traffic jam” expresses negative sentiment in the sentence “I was stuck in a traffic jam on the elevated for 2 h.” But in the domain of transportation, the phrase “traffic jam” in the sentence “Bread and water are essential terms in traffic jams” is without any sentiment. The most common method is to use the domain-specific data samples to classify the text in this domain. However, text sentiment analysis based on machine learning relies on sufficient labeled training data. Aiming at the problem of sentiment classification of news text data with insufficient label news data and the domain adaptation of text sentiment classifiers, an intelligent model, i.e., transfer learning discriminative dictionary learning algorithm (TLDDL) is proposed for cross-domain text sentiment classification. Based on the framework of dictionary learning, the samples from the different domains are projected into a subspace, and a domain-invariant dictionary is built to connect two different domains. To improve the discriminative performance of the proposed algorithm, the discrimination information preserved term and principal component analysis (PCA) term are combined into the objective function. The experiments are performed on three public text datasets. The experimental results show that the proposed algorithm improves the sentiment classification performance of texts in the target domain.

## Introduction

News media platforms and various social media platforms produce a large amount of content every day, including a large number of comments generated by network users. These views and opinions often potentially express their feelings or sentiments. How to exploit users’ sentimental information and analyze their sentimental tendency from these massive comment data has potential application value in many aspects. For example, government departments analyze users’ attitudes toward specific events and topics, grasp public opinions, and keep abreast of public opinion to make appropriate decisions. Commercial enterprises can keep abreast of the market’s reputation by evaluating the relevant content of goods and services, to further improve the quality of their products and services.

Sentiment analysis, also known as sentiment classification, is the field of study that analyses the opinions, views, and sentimental attitudes that people display in texts (Wen et al., 2020; Birjali et al., 2021). Conventional sentiment classification methods are roughly grouped into sentiment dictionary methods and machine learning methods (Zhang et al., 2018; Ahuja et al., 2019). The classification method based on sentiment dictionary mainly uses a manually collated and constructed sentiment dictionary library. The sentiment score of the text is then calculated according to the predetermined rules. Finally, the results of sentiment classification can be obtained based on the obtained sentiment score. There are several obvious shortcomings in the classification method based on sentiment dictionaries. The accuracy of classification results depends on the size of the whole sentiment dictionary resources. When the number of dictionary resources is not large enough, the accuracy of classification results is often not high. In addition, such methods are also difficult to deal with the implied sentimental content. The core of the machine learning methods is the effective feature extraction and classifier. By constructing the feature set, the classifier is trained on the feature set of the training set, and the sentiment label is output for the unlabeled text. In recent years, the commonly used algorithms for text sentiment analysis and text classification include deep learning (Chen et al., 2020; Zhang et al., 2020), decision tree (Almunirawi and Maghari, 2016), support vector machine (Ahmad et al., 2017), sparse representation (Unnikrishnan et al., 2019; Gu et al., 2020), KNN classifier (Bozkurt et al., 2019), Naïve Bayes (Alshamsi et al., 2020), fuzzy logic (Chaturvedi et al., 2019), and extreme learning machine (Lauren et al., 2018; Waheeb et al., 2020).

However, machine learning methods depend on a large number of high-quality label texts, and the manually labeled samples obviously cannot fully meet the needs of sentiment analysis. The expression of sentiment in a text is closely related to the described semantic concept (domain), and the description of sentiment in different domains differs significantly. The distribution of data characteristics in different fields is also different. In this case, directly using the classifiers trained in other domains for text sentiment analysis will lead to the poor adaptability problem. For example, the word “unpredictable” expresses the positive sentiment in literary and artworks, such as “This movie is both exciting and unpredictable! The film is worth seeing!” but in the field of electronic, the word “unpredictable” expresses the negative sentiment, such as “Even if the power is maintained, it is unpredictable. It’s terrible!” As another example, the words “excellent” and “horrible” appear in both domains of electronic and literary works, but “high resolution” and “run fast” rarely appear in the field of literary works, and “printed well” rarely appears in the field of electronic products. Therefore, it is inappropriate to directly use the sentiment classifier trained in the electronic domain to predict the sentiment of comments in the literary works domain.

Recently, the research on cross-domain sentiment classifiers using transfer learning technology becomes a research hotspot (Meng et al., 2019). Transfer learning is a machine learning technology that extracts knowledge from the source domain (the similar but different domain) and applies it to the target domain (the current domain) (Jiang et al., 2020, 2021). The common transfer learning algorithms include sample-based algorithms and feature space-based algorithms (Fu and Liu, 2021; Zhao et al., 2021). The former includes feature selection and feature projection. The sample-based algorithm mainly selects the samples that are valuable for the classification of the target domain from the source domain. To reduce the impact of negative transfer, Gui et al. (2015) developed a negative transfer detection algorithm. This algorithm detected multiple quality levels and retained the high-quality samples. Chen et al. (2010) developed a combined feature level and instance level transfer learning algorithm. This algorithm combined the samples from different domains by evaluating the classification performance in the target domain. Tian et al. (2019) developed an instance-based algorithm for imbalanced cross-linguistic viewpoint analysis. This algorithm translated other relevant markup datasets into the target domain as the supplementary training data.

Feature space-based transfer learning methods include feature selection and feature projection (Xia et al., 2013). The purpose of feature selection is to find the shared features between different domains through some strategies; and then use these features for knowledge transfer. The feature projection strategy maps the features of each domain into a shared feature representation space and establishes the association between the features of each domain. The difference between feature projection and feature selection is that the projected features are not among the original features, but new features. Wu and Tan (2011) developed a two-stage transfer learning sentiment classification algorithm. In the “bridge-building” stage, a bridge between different domains was built to obtain some of the most reliable labeled texts in the target domain. In the “structure following” stage, the internal structure was adopted to label the texts by using the obtained reliable texts. Du et al. (2020) developed a Wasserstein-based transfer network. This algorithm also used a recurrent neural network to capture useful features. López et al. (2019) developed an evolutionary ensemble algorithm for text sentiment analysis. This algorithm was built on a set of evolutionary algorithms. The optimization of the model was achieved by optimizing each sub-algorithm. Wang et al. (2018) developed a measure index called sentiment-related index to measure associations between different domains, and then used this index as a bridge between different domains of features. Tang et al. (2021) developed a graph domain adversarial transfer network for text sentiment classification. This algorithm used a gradient reversal layer to obtain the domain-invariant text features and adopted a projection mechanism to obtain the domain-independent feature representations. Fei et al. (2020) developed a deep learning structure-based transfer learning algorithm, which combined the cross-entropy and weighted for word into the recurrent neural network framework.

Dictionary learning plays a key role in sparse representation and low-rank modeling. The basic operation in sparse representation is called “sparse coding,” which involves reconstructing the data representation using a sparse set of constructive blocks (called “atoms”), and the atoms are clustered in a structure called a “dictionary” (Gu et al., 2021; Ni et al., 2021). Therefore, the dictionary learning algorithm has data self-adaptability, which aims to learn a series of basic atoms from the dataset to linearly approximate the given data. Since dictionary learning learns a set of atoms to obtain discriminative feature representation, it performs well in classification tasks. In this study, I develop a transfer learning discriminative dictionary learning (TLDDL) algorithm for cross-domain text sentiment classification. Considering the distribution difference between different domains, I adopt the subspace technology to project all samples into a subspace. In the common subspace, a domain-invariant dictionary is built to connect different domains. Moreover, the discrimination information preserved term and PCA term are embedded into the objective function, so that the performance of TLDDL for classification tasks across domains is enhanced. TLDDL learns all parameters in an alternating iteration manner. By comparing several non-transfer learning and transfer learning algorithms on toutiao-text (Zhang et al., 2021), Chinese Weibo (Bai et al., 2020), and Amazon review (Pan et al., 2010) datasets, one can draw that the TLDDL algorithm is efficient.

The rest of this paper is organized as follows: the next section presents dictionary learning. The proposed TLDDL algorithm and its optimization are described in section “Transfer Learning Discriminative Dictionary Learning Algorithm”. Experimental results are given in section “Experiment.” Finally, section “Conclusion” concludes the paper.

## Backgrounds

Given a data matrix **Y**, the dictionary learning algorithm attempts to learn the dictionary **D** so that the data **Y** can be approximately linearly expressed as **Y**≈**DS**. Dictionary learning can be represented as,


(1) $$\begin{array}{c} \arg\,\min_{D,S}∥\text{Y}-{\mathrm{DS}∥}_{F}^{2},\\ s.t.\forall i,{∥s_{i}∥}_{0}\leq T_{i}, \end{array}$$

where *T*_*i*_ is the given threshold. **S** is the sparse coding matrix.

The constraints are used to constrain the complexity of the dictionary matrix. Dictionary learning can also be represented as the following optimization problem, where the constraints are replaced as well as the objective function,


(2) $$\begin{array}{c} \min_{D,S}{∥{\text{s}}_{i}∥}_{0},i=1,\ldots ,n\\ s.t.∥\text{Y}-\text{DS}{∥}_{F}^{2}<\varepsilon ,\\ {∥d_{j}∥}_{2}^{2}<1,j=1,\ldots ,K \end{array}$$

Where ∥⋅∥_0_ represents the 0 norm of the vector. *n* and *K* are the number of training samples and atoms, respectively. It is difficult to optimize the 0 norm in Equations 1, 2, so the 1 norm is commonly used in dictionary learning. For the above optimization problem, after the transformation of the Lagrange equation, Equation 1 can be represented as follows,


(3) $$\begin{array}{c} \min_{D,S}∥\text{Y}-{\mathrm{DS}∥}_{F}^{2}+\lambda \,{∥s_{i}∥}_{1},i=1,\ldots ,n\\ s.t.{∥d_{j}∥}_{2}^{2}<1,j=1,\ldots ,K \end{array}$$

It is easily can be seen that dictionary learning minimizes the reconstruction error $∥\text{Y}-\text{DS}{∥}_{F}^{2}$, while the 1 norm of sparse coding **s**_*i*_ is as small as possible, that is, the sparsity of **S** is as strong as possible. λ is the trade-off parameter. The optimization process of dictionary learning generally includes two alternating iterative steps, one is to solve sparse coding **S**, and the other is to solve dictionary **D**. The optimization problem of sparse coding **S** is


(4) $$\min_{D,S}∥\text{Y}-{\mathrm{DS}∥}_{F}^{2}+\lambda \,{∥s_{i}∥}_{1},i=1,\ldots ,n$$

And the optimization problem of dictionary **D** is


(5) $$\begin{array}{c} \min_{D,S}∥\text{Y}-{\mathrm{DS}∥}_{F}^{2},\\ s.t.{∥d_{j}∥}_{2}^{2}<1,j=1,\ldots ,K \end{array}$$

By alternately optimizing the above problems until convergence, the optimal **D** and **S** can be obtained. For the sample **y** on the test set, the corresponding sparse coding can be obtained by solving Equation 4, and then the classification is performed using the obtained sparse coding.

## Transfer Learning Discriminative Dictionary Learning Algorithm

### Objective Function

It is known that when there are few domain-invariant features between different domains, the cross-domain classification performance will decline. To extract as sufficient domain-invariant features as possible and reduce their differences between different domains, the TLDDL algorithm uses the dictionary learning model to learn a domain-invariant dictionary as a bridge to realize the association of the two domains. The source domain is **Y**_*s*_ = {**y**_*s*,1_,**y**_*s*,2_,…,**y**_*s*,*n*_*s*__}, and the target domain is **Y**_*t*_ = {**y**_*t*,1_,**y**_*t*,2_,…,**y**_*t*,*n*_*t*__}, where *n*_*s*_ and *n*_*t*_ are the number of the source domain and the target domain, respectively.

Firstly, I use the *k*-nearest neighbor knowledge to represent the local structure and label information of the original data. I think that if the sample **y**_*j*_ is in the *k*-nearest neighbor of its same-class sample **y**_*i*_, then the corresponding sparse coding coefficients **s**_*i*_ and **s**_*j*_ for **y**_*i*_ and **y**_*j*_ should also be closer to each other. On the other hand, it is necessary to minimize the within-class variance and maximize the between-class separability for the sparse coding coefficients.

Given the training samples **y**_*i*_ and **y**_*j*_, the following within-class graph matrix *E*^*wit*^ and between-class graph matrix ${\text{E}}_{s}^{b\,e\,t}$ are defined as


(6) $$e_{i\,j}^{\text{wit}}=\left\{ \begin{array}{c} e\,\left({\text{y}}_{i},{\text{y}}_{j}\right),i\,f\,{\text{y}}_{i}\in N_{w\,i\,t}\,\left({\text{y}}_{j}\right)\,o\,r\,{\text{y}}_{j}\in N_{w\,i\,t}\,\left({\text{y}}_{i}\right)\\ 0,o\,t\,h\,e\,r\,w\,i\,s\,e \end{array}\right.$$

(7) $$e_{i\,j}^{b\,e\,t}=\left\{ \begin{array}{c} -e\,\left({\text{y}}_{i},{\text{y}}_{j}\right),i\,f\,{\text{y}}_{i}\in N_{b\,e\,t}\,\left({\text{y}}_{j}\right)\,o\,r\,{\text{y}}_{j}\in N_{b\,e\,t}\,\left({\text{y}}_{i}\right)\\ 0,o\,t\,h\,e\,r\,w\,i\,s\,e \end{array}\right.$$

where *e*(**y**_*i*_,**y**_*j*_) = *exp*⁡(−∥**y**_*i*_−**y**_*j*_∥^2^/θ), θ is the tune parameter. *N*_*wit*_(**y**_*i*_) is the *k*-nearest neighbors of **y**_*i*_ belonging to the same class, and *N*_*bet*_(**y**_*i*_) is the *k*-nearest neighbors of **y**_*i*_ belonging to the different class.

Considering the within-class minimizing and between-class maximizing of sparse coding coefficients, I define the following discrimination information preserved term *F*_1_(**S**_*t*_) in the source domain,


(8) $$\begin{array}{c} F_{1}\,\left({\text{S}}_{s}\right)={\text{min}}_{S_{s}}\frac{1}{2}\,\sum_{i=1}^{n_{s}}\sum_{j=1}^{n_{s}}{∥{\text{s}}_{s,i}-{\text{s}}_{s,j}∥}_{2}^{2}\,\left({\text{E}}_{s}^{w\,i\,t}-{\text{E}}_{s}^{b\,e\,t}\right)\\ =T\,r\,\left({\text{S}}_{s}\,{\text{L}}_{s}^{w\,i\,t}\,{\text{S}}_{s}^{T}\right)-T\,r\,\left({\text{S}}_{s}\,{\text{L}}_{s}^{b\,e\,t}\,{\text{S}}_{s}^{T}\right)\\ =T\,r\,\left({\text{S}}_{s}\,{\text{Q}}_{s}\,{\text{S}}_{s}^{T}\right), \end{array}$$

where ${\text{Q}}_{s}={\text{L}}_{s}^{w\,i\,t}-{\text{L}}_{s}^{b\,e\,t}$, *Tr*(⋅) is the trace operator.

Similarly, I can obtain the discrimination information preserved term *F*_1_(*S*_*t*_) in the target domain,


(9) $$\begin{array}{c} F_{1}\,\left({\text{S}}_{t}\right)=\underset{S_{t}}{\text{min}}\frac{1}{2}\,\sum_{i=1}^{n_{t}}\sum_{j=1}^{n_{t}}{∥{\text{s}}_{t,i}-{\text{s}}_{t,j}∥}_{2}^{2}\,\left({\text{E}}_{t}^{w\,i\,t}-{\text{E}}_{t}^{b\,e\,t}\right)\\ =T\,r\,\left({\text{S}}_{t}\,{\text{L}}_{t}^{w\,i\,t}\,{\text{S}}_{t}^{T}\right)-T\,r\,\left({\text{S}}_{t}\,{\text{L}}_{t}^{b\,e\,t}\,{\text{S}}_{t}^{T}\right)\\ =T\,r\,\left({\text{S}}_{t}\,{\text{Q}}_{t}\,{\text{S}}_{t}^{T}\right), \end{array}$$

where ${\text{Q}}_{t}={\text{L}}_{t}^{w\,i\,t}-{\text{L}}_{t}^{b\,e\,t}$.

When reconstructing the projection space, the proposed transfer learning algorithm not only needs to establish potential connections between multiple domains in the projection space, but also transfers the domain-invariant information from the source domain to the target domain. Meanwhile, TLDDL should also retain the identification information of label samples and maintain the discriminative information in the projection space. To achieve this goal, I establish the principal component analysis (PCA) term of the projection matrix. Based on the PCA criterion, the discrimination information in the original space will be retained in the projection space. The PCA terms of the projection matrix on the source and target domains are represented as


(10) $$F_{2}\,\left({\text{P}}_{s}\right)=\max_{P_{s}}T\,r\,\left({\text{P}}_{s}\,{\text{Y}}_{s}\,{\text{Y}}_{s}^{T}\,{\text{P}}_{s}^{T}\right),$$

(11) $$F_{2}\,\left({\text{P}}_{t}\right)=\max_{P_{t}}T\,r\,\left({\text{P}}_{t}\,{\text{Y}}_{t}\,{\text{Y}}_{t}^{T}\,{\text{P}}_{t}^{T}\right),$$

where **P**_*s*_,**P**_*t*_ ∈ **R**^*p*×*d*^, *p* and *d* are the dimensions of the projection subspace and original feature space, respectively.

Based on the traditional dictionary learning algorithm, TLDDL combined the discriminative information preserved terms and PCA terms of the projection matrix together, and builds a domain-invariant dictionary between different domains. The objective function of TLDDL is


(12) $$\begin{array}{ccc} \min_{D,P_{s},P_{t},S_{s},S_{t}}{∥P_{s}\,Y_{s}^{T}-D\,S_{s}∥}_{2}^{2}+{∥P_{t}\,Y_{t}^{T}-D\,S_{t}∥}_{2}^{2}+\delta \,T\,r\,\left(S_{s}\,Q_{s}\,S_{s}^{T}\right) & & \\ +\delta \,T\,r\,\left(S_{t}\,Q_{t}\,S_{t}^{T}\right) & & \\ -\gamma \,T\,r\,\left(P_{s}\,Y_{s}\,Y_{s}^{T}\,P_{s}^{T}\right)-\gamma \,T\,r\,\left(P_{t}\,Y_{t}\,Y_{t}^{T}\,P_{t}^{T}\right)+\beta \,{∥S_{s}∥}_{2}^{2} & & \\ +\beta \,{∥S_{t}∥}_{2}^{2}, & & \\ s.t.{∥d_{i}∥}_{2}^{2}\leq 1, & & \\ P_{s}^{T}\,P_{s}=I, & & \\ P_{t}^{T}\,P_{t}=I, & & \end{array}$$

where δ, γ and β are trade off parameters.

For Equation 12, the first and second terms inherit the dictionary learning algorithm and are used to reconstruct the data in the source and target domains. These two terms are also domain-invariant dictionary learning terms to realize the connection of knowledge between different domains. The third and fourth terms are discriminative information preserved terms. The fifth and sixth terms are PCA terms of the projection matrices. The last two terms are the regularization terms of the sparse coding matrices.

### Optimization

Let $\text{P}=\left[{\text{P}}_{s},{\text{P}}_{t}\right],\;Y=\left[\begin{array}{cc} {\text{Y}}_{s}^{T} & 0\\ 0 & {\text{Y}}_{t}^{T} \end{array}\right],S=\left[\begin{array}{cc} {\text{S}}_{s} & 0\\ 0 & {\text{S}}_{t} \end{array}\right],\;Q=\left[\begin{array}{cc} {\text{Q}}_{s} & 0\\ 0 & {\text{Q}}_{t} \end{array}\right],$ Equation 12 can be simplified as,


(13) $$\begin{array}{c} \min_{D,P,S}{∥{\text{PY}}^{T}-\text{DS}∥}_{F}^{2}+T\,r\,\left(\text{S}\,\left(\delta \,\text{Q}+\beta \,\text{I}\right)\,{\text{S}}^{T}\right)-\gamma \,T\,r\,\left({\text{PYY}}^{T}\,{\text{P}}^{T}\right),\\ s.t.{∥{\text{d}}_{i}∥}_{2}^{2}\leq 1,\forall i\\ P^{T}\,\text{P}=\text{I}, \end{array}$$

The alternating iteration method is used to optimize variables {**P**,**S**,**D**}. In the optimization process, other variables are fixed and only one variable is optimized.

Fixed{**S**,**D**}, the optimization problem of parameter **P** can be written as,


(14) $$\begin{array}{c} \min_{P}{∥{\text{PY}}^{T}-\text{DS}∥}_{F}^{2}-\gamma \,T\,r\,\left({\text{PYY}}^{T}\,{\text{P}}^{T}\right),\\ s.t.{\text{PP}}^{T}=\text{I}. \end{array}$$

Following the Proposition (2) in Shekhar et al. (2013), let


(15) $$\text{P}={\left(\mathrm{YW}\right)}^{T},$$

(16) D=PYC,

where **W** ∈ **R**^(*n*_*s*_ + *n*_*t*_)×*p*^, **C** ∈ **R**^(*n*_*s*_ + *n*_*t*_)×*K*^.

Equation 14 can be written as,


(17) $$\begin{array}{c} \min_{W}{∥{\text{W}}^{T}\,\text{M}\,\left(\text{I}-\text{CS}\right)∥}_{F}^{2}-\gamma \,T\,r\,\left({\text{W}}^{T}\,{\text{MM}}^{T}\,\text{W}\right),\\ s.t.{\text{WMW}}^{T}=\text{I}. \end{array}$$

where **M** = **Y**^*T*^**Y**.

Then **W** has a closed-form solution as,


(18) $$\text{W}=\Theta \,{\text{V}}^{-1/2}\,{\text{U}}^{T},$$

where **M** = Θ**V**Θ^*T*^, and **U** can be obtained as,


(19) $$\begin{array}{c} \min_{U}T\,r\,\left({\text{U}}^{T}\,{\text{H}}^{T}\,U\right),\\ s.t.{\text{U}}^{T}\,\text{U}=\text{I}, \end{array}$$

where **H** = **V**^1/2^Θ^*T*^((**I**−−**CS**)(**I**−−**CS**)^*T*^−γ**I**)Θ**V**^1/2^. Due to the orthonormality condition on **U**, Equation 19 has a closed-form solution. Therefore, **P** can then be updated by Equation 15.

Fixed{**P**,**S**}, the optimization problem of parameter **D** can be written as,


(20) $$\begin{array}{c} \min_{D}{∥\text{PY}-\text{DS}∥}_{F}^{2},\\ s.t.{∥{\text{d}}_{i}∥}_{2}^{2}\leq 1. \end{array}$$

Using the Lagrange dual method, **D** can be obtained as,


(21) $$\text{D}=\left({\text{PYS}}^{T}\right)\,{\left({\text{SS}}^{T}+\Delta \right)}^{-1},$$

where Δ is a diagonal matrix.

Fixed {**P**,**D**}, the optimization problem of parameter **S** can be written as,


(22) $$\min_{S}{∥{\text{PY}}^{T}-\text{DS}∥}_{F}^{2}+T\,r\,\left(\text{S}\,\left(\delta \,\text{Q}+\beta \,\text{I}\right)\,{\text{S}}^{T}\right),$$

By setting the derivative of **S** to zero, I get,


(23) $${\text{D}}^{T}\,\text{DS}+\text{S}\,\left(\delta \,\text{Q}+\beta \,\text{I}\right)={\text{D}}^{T}\,\text{PY}.$$

In this study, Equation 23 is solved by the Bartels-Stewart method (Kleinman and Rao, 2003).

The above optimization steps are summarized in Algorithm 1.

**ALGORITHM 1:** Algorithm 1 the TLDDL algorithm.

Input: Training data **Y**_*s*_ and **Y**_*t*_;
Output: dictionary **D**, projection matrix **P**_*s*_ and **P**_*t*_
Step 1. Initialize **D** and **S** using the K-SVD algorithm (Aharon et al., 2006);
Step 2. Compute the matrices **E**^*wit*^ and ${\text{E}}_{s}^{b\,e\,t}$ *via* Equation 6, 7;
Step 3. Learn **P** with fixed {**S**,**D**} *via* Equations 14–19;
Step 4. Learn **D** with fixed {**P**,**S**} *via* Equation 21;
Step 5. Learn **S** with fixed {**P**,**D**} *via* Equation 23;
Step 6. Go to Step 3 until the convergence or reaching the maximum number of iterations
Step 7. Output the dictionary **D**, projection matrix **P**_*s*_ and **P**_*t*_.

### Test

For an unlabeled test text **y**^*test*^, according to the obtained optimal projection matrix **P**_*t*_ and dictionary **D**, its sparse coding coefficient s^*^ can be solved by the following problem,


(24) $$\min_{s^{*}}{∥{\text{P}}_{t}\,{\left({\text{y}}^{t\,e\,s\,t}\right)}^{T}-{\text{Ds}}^{*}∥}_{2}^{2}+\beta \,{∥{\text{s}}^{*}∥}_{2}^{2}.$$

I can obtain s^*^ as follows,


(25) $${\text{s}}^{*}={\left({\text{DD}}^{T}+\beta \,\text{I}\right)}^{-1}\,{\text{D}}^{T}\,{\text{P}}_{t}.$$

Then, I compute the reconstruction error of **y**^*test*^ on the dictionary *D* = [**D**_1_,**D**_2_,…,**D**_*J*_], where *J* is the number of sample classes. The reconstruction error of **y**^*test*^ on the *j*th sub dictionary **D**_*j*_ can be computed as follows,


(26) $${\text{r}}_{j}\,\left({\text{y}}^{t\,e\,s\,t}\right)={∥{\text{y}}^{t\,e\,s\,t}-{\text{D}}_{j}\,{\text{s}}_{j}^{*}∥}_{2}^{2},$$

where ${\text{s}}_{j}^{*}$ is the sparse coding coefficient of **y**^*test*^ on the *D*_*j*_. Finally, the class label of **y**^*test*^ is the class with the smallest reconstruction error, i.e.,


(27) $$l\,a\,b\,e\,l\,\left({\text{y}}^{t\,e\,s\,t}\right)=\min_{j}{\text{r}}_{j}\,\left({\text{y}}^{t\,e\,s\,t}\right).$$

## Experiment

### Datasets and Experiment Setup

The experiment adopts the Chinese corpus (including toutiao-text and Chinese Weibo datasets) to verify the proposed TLDDL algorithm. I select 13 categories in Chinese corpus includes: Story (Sto), Culture (Cul), Entertainment (Ent), Sports (Spo), Finance (Fin), House (Hou), Car, Education (Edu), Technology (Tec), Military (Mil), World (Wor), Agriculture (Agr), and Game (Gam). Each category represents a domain. In addition, the experiment adopts the English multi-domain dataset Amazon review dataset, which is often used in cross-domain sentiment classification. Amazon review dataset includes reviews of DVD, book (Boo), electronic (Ele), kitchen and household appliance (Kit). Each product also represents a domain. To facilitate comparison with existing methods, 2,000 comments are selected respectively, including 1,000 positive comments and 1,000 negative comments. I use 80% texts in the source domain and 10% texts in the target domain as the training dataset, and the rest of the texts in the target domain are used for testing.

I investigate the performance of TLDDL compared with several algorithms on two text corpora. For comparison, the K-SVD algorithm (Aharon et al., 2006) is used as the baseline of the proposed algorithm. The size of dictionary in K-SVD is set to 300. In addition, six transfer learning algorithms are used, including: ARTL (Long et al., 2014), DMTTL (Zheng et al., 2019), SMITL (Liu et al., 2018), WAAR (Jia et al., 2018), and SFA (Pan et al., 2010). In ARTL, the parameter λ is set in the grid {10^−2^,10^−1^,…,10^2^}, the parameter γ is set in the grid {0.01,0.05,0.1,0.5,1.5,10}, and the parameter σ is set in the grid {0.01, 0.02, 0.1, 0.5, 1, 2, 5}. In DMTTL, the parameters λ,λ_*s*_,λ_*t*_ are set in the grid {10^−4^,10^−3^,…,10^3^}, γ is set in the grid {10^2^,5×10^2^,…,2×10^4^}. In SMITL, the Gaussian kernel is used, and the kernel and penalty parameters are set in the grid {10^−4^,10^−3^,…,10^3^}. In WAAR, the parameter *l* is set to 600, min-support = 0.014, min-confidence = 0.08, and ε = 0.005. In SFA, the parameters *l*, *k* and γ are set to 500, 100 and 0.6, respectively. In the proposed TLDDL, the subspace dimension and the size of dictionary are set to 500 and 300, respectively. The parameters δ_*s*_, δ_*t*_, γ_*s*_, γ_*t*_, β_*s*_ and β_*t*_are set in the grid {0.01, 0.05,…, 2}. In the experiments, the dimension of the text word vectors is set to 300. I take the first 100 text units in each text. Following (Meng et al., 2019), a convolutional neural network composed of five layers is used to extract the text features. The mini-batch size is set to 16, and the number of convolution kernels is set to 256. Finally, these vectors are set to 768 dimensions when I treat them into the training model. All the experiments are executed in Matlab 2018a environment. I repeat the experiments five times. The performances of experimental results are generally evaluated based on accuracy, precision, recall, and F1-score.

### Experiments on Chinese Corpus

Tables 1–4 show the classification results on Chinese corpus based on accuracy, precision, recall, and F1-score, respectively. For example, task “Stor→Cul” indicates that the source domain is “Story” and the target domain is “Culture.” In the respect of classification accuracy, the best average accuracy obtained by TLDDL is 81.23%, followed by SFA, and its average accuracy is 79.24%. The non-transfer learning algorithm K-SVD is the lowest. In the respect of precision, recall, and F1-score, all transfer learning algorithms are always better than the non-transfer learning algorithm K-SVD in each classification task. The proposed TLDDL algorithm also obtains the best performance. Compared with the second-best algorithm, the average precision, recall, and F1 score exceed 1.99, 1.97, and 1.96%, respectively. Therefore, the generalization ability of the proposed TLDDL algorithm is higher. The experimental results indicate that under the framework of the dictionary learning algorithm, TLDDL uses the projection technology to reduce the differences between different domains, and builds a domain-invariant dictionary to establish a bridge between the related domains. The transferring discriminative information of the source domain to the target domain can improve the cross-domain classification performance.

**TABLE 1:** Accuracy results for each cross-domain task on Chinese corpus.

Tasks	K-SVD	ARTL	DMTTL	SMITL	WAAR	SFA	TLDDL
Sto→Cul	62.31	66.18	66.64	66.35	68.89	68.63	**70.64**
Sto→Ent	77.69	80.02	81.01	81.14	83.03	83.59	**85.58**
Cul→Ent	77.05	80.54	80.90	81.55	82.03	83.06	**85.05**
Cul→Edu	78.72	82.37	81.39	83.85	84.87	84.79	**86.76**
Ent→Spo	85.07	89.34	88.40	90.99	91.85	91.98	**93.89**
Ent→Edu	77.15	80.13	81.08	81.91	83.99	83.59	**85.51**
Spo→Wor	64.00	67.62	67.64	68.08	69.06	70.01	**72.01**
Spo→Gam	82.04	85.46	85.02	86.37	87.86	88.02	**90.03**
Fin→Car	85.08	88.59	88.59	89.94	90.15	91.12	**93.02**
Fin→Agr	78.33	81.89	82.08	81.76	83.22	84.40	**86.42**
Hou→Wor	63.11	67.29	67.67	67.15	69.13	69.72	**71.77**
Hou→Fin	58.04	60.29	61.13	62.08	63.71	63.93	**66.05**
Car→Tec	79.18	83.13	83.40	84.84	85.00	85.82	**87.87**
Car→Gam	82.28	85.78	86.82	87.61	87.33	88.22	**90.21**
Edu→Tec	79.02	82.14	82.89	83.78	84.04	85.34	**87.32**
Edu→Sto	61.29	65.54	65.91	65.00	67.70	67.45	**69.43**
Tec→Wor	63.30	67.32	67.56	68.43	69.47	69.82	**71.87**
Tec→Mil	78.07	81.65	81.09	82.97	83.28	84.10	**86.02**
Mil→Sto	60.19	64.39	64.85	65.04	66.93	66.85	**68.97**
Mil→Agr	75.10	79.58	79.28	80.94	81.16	82.05	**84.05**
Wor→Mil	78.60	81.21	82.92	83.97	84.67	84.67	**86.66**
Wor→Cul	63.06	66.65	66.12	67.65	68.60	69.00	**71.03**
Agr→Fin	58.29	62.91	62.06	63.74	64.18	64.76	**66.78**
Agr→Sto	60.29	64.39	64.18	65.98	66.10	66.87	**68.88**
Gam→Spo	85.05	89.56	89.93	89.10	91.72	91.43	**93.47**
Gam→Car	84.17	88.07	88.85	89.80	90.94	90.92	**92.79**
Mean	72.94	76.62	76.82	77.69	78.81	79.24	**81.23**

*The best classification results are indicated in bold in Table.*

**TABLE 2:** Precision results for each cross-domain task on Chinese corpus.

Tasks	K-SVD	ARTL	DMTTL	SMITL	WAAR	SFA	TLDDL
Stor→Cul	68.13	72.23	72.69	72.26	75.03	74.64	**76.68**
Stor→Ent	77.74	80.09	81.05	81.34	82.84	83.58	**85.54**
Cul→Ent	76.81	80.31	80.81	81.51	82.34	83.17	**85.37**
Cul→Edu	76.67	80.54	79.51	81.92	82.64	82.64	**84.44**
Ent→Spo	85.15	89.10	88.47	90.90	91.70	92.04	**94.10**
Ent→Edu	75.09	78.09	79.16	79.89	81.99	81.54	**83.41**
Spo→Wor	64.07	67.61	67.38	68.02	69.14	69.92	**71.96**
Spo→Gam	84.25	87.46	87.17	88.46	90.04	90.09	**92.35**
Fin→Car	85.29	88.53	88.48	90.06	90.15	91.01	**93.22**
Fin→Agr	78.34	81.98	82.04	81.87	83.25	84.66	**86.38**
Hou→Wor	63.03	67.21	67.41	67.21	69.11	69.79	**71.81**
Hou→Fin	57.99	60.35	61.15	62.14	63.57	63.92	**66.03**
Car→Tec	79.01	83.19	83.41	84.59	85.10	85.94	**87.70**
Car→Gam	84.34	87.67	88.94	89.78	89.48	90.36	**92.15**
Edu→Tec	78.95	82.21	82.98	83.75	84.01	85.56	**87.50**
Edu→Stor	59.32	63.46	63.67	62.76	65.85	65.47	**67.20**
Tec→Wor	63.17	67.02	67.58	68.28	69.52	70.01	**71.71**
Tec→Mil	81.03	84.71	84.17	85.94	86.34	87.14	**89.17**
Mil→Sto	58.34	62.22	62.95	63.25	65.15	64.84	**67.03**
Mil→Agr	75.16	79.26	78.96	80.94	80.95	82.03	**83.87**
Wor→Mil	81.47	84.09	85.92	87.06	87.61	87.65	**89.88**
Wor→Cul	62.96	66.71	66.30	67.53	68.87	68.95	**71.28**
Agr→Fin	58.43	62.96	61.95	63.67	64.40	64.94	**67.02**
Agr→Sto	60.14	64.39	64.14	65.99	65.94	66.74	**68.88**
Gam→Spo	85.05	89.45	89.71	89.01	91.83	91.61	**93.27**
Gam→Car	84.10	88.10	88.87	89.82	90.86	90.81	**92.96**
Mean	73.23	76.88	77.11	78.00	79.14	79.58	**81.57**

*The best classification results are indicated in bold in Table.*

**TABLE 3:** Recall results for each cross-domain task on Chinese corpus.

Tasks	K-SVD	ARTL	DMTTL	SMITL	WAAR	SFA	TLDDL
Stor→Cul	76.10	79.95	80.70	80.53	82.62	82.69	**84.61**
Stor→Ent	76.63	79.09	80.00	80.12	82.04	82.56	**84.43**
Cul→Ent	75.99	79.63	79.75	80.56	80.74	81.87	**84.28**
Cul→Edu	77.62	81.38	80.41	82.59	83.92	83.91	**85.58**
Ent→Spo	83.88	88.55	87.18	89.76	90.74	91.10	**92.86**
Ent→Edu	76.28	79.21	80.30	80.84	82.79	82.62	**84.49**
Spo→Wor	61.09	64.63	64.43	65.19	66.20	66.85	**69.25**
Spo→Gam	77.01	80.38	80.06	81.25	83.13	82.95	**85.16**
Fin→Car	84.25	87.40	87.54	89.15	88.98	90.17	**92.06**
Fin→Agr	77.36	80.86	81.11	80.76	82.14	83.56	**85.46**
Hou→Wor	62.23	66.47	66.83	66.16	68.21	68.91	**70.53**
Hou→Fin	57.76	60.59	61.32	62.32	63.70	64.13	**66.11**
Car→Tec	77.91	82.43	82.39	83.81	83.97	84.95	**86.62**
Car→Gam	77.01	80.72	81.74	82.68	82.43	82.99	**85.04**
Edu→Tec	77.97	81.22	81.87	82.57	82.90	84.04	**86.39**
Edu→Stor	63.28	67.53	67.81	67.26	69.59	69.59	**71.54**
Tec→Wor	60.30	64.38	64.74	65.32	66.53	66.89	**68.70**
Tec→Mil	74.04	77.75	77.00	79.16	79.47	80.37	**81.97**
Mil→Sto	61.94	66.26	66.68	67.17	68.95	68.81	**71.18**
Mil→Agr	74.02	78.74	78.24	79.96	80.04	81.32	**82.78**
Wor→Mil	74.45	77.42	79.09	80.00	80.47	80.61	**82.75**
Wor→Cul	77.13	80.56	80.10	81.83	82.70	83.04	**85.02**
Agr→Fin	58.52	63.11	61.89	63.92	64.17	64.64	**66.82**
Agr→Sto	59.41	63.66	63.29	64.99	65.33	65.84	**67.77**
Gam→Spo	83.90	88.56	88.85	87.92	90.78	90.26	**92.36**
Gam→Car	83.24	87.01	87.65	88.86	90.07	89.83	**91.87**
Mean	72.67	76.44	76.58	77.49	78.56	79.02	**80.99**

*The best classification results are indicated in bold in Table.*

**TABLE 4:** F1-score results for each cross-domain task on Chinese corpus.

Tasks	K-SVD	ARTL	DMTTL	SMITL	WAAR	SFA	TLDDL
Stor→Cul	72.50	75.91	76.44	76.41	79.09	78.62	**80.55**
Stor→Ent	77.41	79.44	80.54	80.62	82.76	83.06	**84.76**
Cul→Ent	76.23	79.90	80.44	80.93	82.03	82.48	**84.70**
Cul→Edu	77.26	80.97	80.19	82.34	83.26	83.34	**84.66**
Ent→Spo	84.53	88.76	87.83	89.94	91.34	91.97	**93.48**
Ent→Edu	75.83	78.98	79.86	80.40	82.17	81.77	**84.22**
Spo→Wor	62.63	66.18	65.75	66.31	67.89	68.52	**70.85**
Spo→Gam	80.71	83.54	83.34	84.83	86.72	86.40	**88.45**
Fin→Car	84.83	87.72	87.91	89.61	89.69	90.53	**92.68**
Fin→Agr	78.08	81.22	81.50	81.19	82.59	84.54	**86.02**
Hou→Wor	62.93	67.18	66.99	66.93	68.53	69.51	**71.01**
Hou→Fin	57.92	60.16	61.20	62.57	63.61	63.70	**66.05**
Car→Tec	78.47	83.20	83.05	84.10	84.37	85.68	**86.75**
Car→Gam	80.37	84.15	85.17	86.03	86.00	86.85	**88.81**
Edu→Tec	78.88	81.77	82.27	82.99	83.17	84.90	**86.93**
Edu→Stor	61.08	65.37	65.34	64.68	67.43	67.60	**69.38**
Tec→Wor	61.85	65.72	65.87	66.72	68.24	68.40	**70.33**
Tec→Mil	77.28	81.46	80.43	82.39	82.87	83.94	**85.73**
Mil→Sto	60.14	64.29	64.76	64.87	66.86	66.75	**69.22**
Mil→Agr	74.49	78.99	78.66	80.76	80.56	81.64	**83.01**
Wor→Mil	77.81	80.48	82.72	83.75	83.89	84.24	**86.41**
Wor→Cul	69.84	73.58	72.86	74.60	75.94	75.60	**78.25**
Agr→Fin	58.47	63.05	61.76	63.93	64.05	64.87	**67.03**
Agr→Sto	59.67	64.21	63.51	65.59	65.88	66.34	**68.44**
Gam→Spo	84.18	89.08	88.98	88.52	91.51	90.65	**93.07**
Gam→Car	83.66	87.58	88.21	89.37	90.50	90.26	**92.22**
Mean	72.96	76.65	76.75	77.71	78.88	79.31	**81.27**

*The best classification results are indicated in bold in Table.*

### Experiments on English Corpus

Tables 5–8 show the classification results on the English corpus based on accuracy, precision, recall, and F1-score, respectively. I can see that all transfer learning algorithms outperform the baseline algorithm K-SVD. Some tasks have high classification performance, such as DVD→Boo and Boo→DVD. The reason for the high classification performance is that the differences between the source domain and the target domain are similar. Some tasks have low classification performance, such as DVD→Ele and DVD→Kit. The reason is that the domain-invariant information transferring from the source domain to the target domain is insufficient due to the great differences between domains. The results in Tables 5–8 show that the TLDDL algorithm obtains the best performance. TLDDL obtains the classification performance 81.26, 81.67, 80.87, and 81.25% in the respect of accuracy, precision, recall and F1-score, respectively. TLDDL is 1.02, 0.94, 0.98, and 1.00% higher than the second-best algorithm in four evaluation indexes. The results indicate that joint learning of projection technology and dictionary learning is an efficient strategy in cross-domain text classification tasks. In addition, the discrimination information preserved and PCA terms are helping to improve the generalization of the classifier.

**TABLE 5:** Accuracy results for each cross-domain task on English corpus.

Tasks	K-SVD	ARTL	DMTTL	SMITL	WAAR	SFA	TLDDL
DVD→Boo	74.44	76.76	77.18	78.00	79.95	81.05	**82.98**
DVD→Ele	70.28	72.44	73.30	73.68	74.65	76.01	**76.78**
DVD→Kit	71.09	73.70	73.79	74.92	75.03	75.67	**76.09**
Boo→DVD	76.32	79.02	79.20	81.02	82.01	82.94	**84.74**
Boo→Ele	71.32	73.41	74.12	75.52	76.29	77.28	**78.07**
Boo→Kit	73.64	76.36	76.64	78.13	77.43	77.67	**78.12**
Ele→DVD	73.04	75.78	75.96	77.56	79.02	80.54	**81.63**
Ele→Boo	70.88	73.88	73.34	75.68	76.67	78.66	**80.33**
Ele→Kit	83.23	85.61	86.32	87.71	85.39	86.37	**87.54**
Kit→DVD	74.73	77.44	77.04	78.77	79.27	80.14	**80.78**
Kit→Boo	82.07	84.53	85.11	86.03	86.02	86.90	**87.28**
Kit→Ele	73.65	75.95	76.59	77.57	78.88	79.67	**80.76**
Mean	74.56	77.07	77.38	78.72	79.22	80.24	**81.26**

*The best classification results are indicated in bold in Table.*

**TABLE 6:** Precision results for each cross-domain task on English corpus.

Tasks	K-SVD	ARTL	DMTTL	SMITL	WAAR	SFA	TLDDL
DVD→Boo	74.75	77.65	77.45	78.57	80.26	81.36	**83.25**
DVD→Ele	71.03	73.02	73.82	73.96	75.28	76.16	**77.12**
DVD→Kit	71.85	73.84	73.89	75.13	75.68	76.32	**76.43**
Boo→DVD	76.51	79.88	79.45	81.02	82.76	83.71	**85.42**
Boo→Ele	72.13	73.93	74.75	75.58	77.15	77.49	**78.40**
Boo→Kit	73.96	76.83	77.02	78.26	77.82	78.57	**78.61**
Ele→DVD	73.15	75.80	76.16	77.77	79.85	81.16	**81.80**
Ele→Boo	71.60	74.16	73.75	75.98	77.42	78.97	**80.90**
Ele→Kit	83.78	85.68	86.84	88.21	85.73	87.06	**88.39**
Kit→DVD	75.53	77.81	77.28	79.26	79.39	80.85	**81.30**
Kit→Boo	82.58	84.90	85.62	86.77	86.54	87.26	**87.67**
Kit→Ele	73.80	76.73	77.05	78.31	79.60	79.86	**80.77**
Mean	75.06	77.52	77.76	79.07	79.79	80.73	**81.67**

*The best classification results are indicated in bold in Table.*

**TABLE 7:** Recall results for each cross-domain task on English corpus.

Tasks	K-SVD	ARTL	DMTTL	SMITL	WAAR	SFA	TLDDL
DVD→Boo	73.86	76.23	77.14	77.30	79.93	80.44	**82.69**
DVD→Ele	69.48	71.69	72.82	73.51	73.98	75.38	**76.14**
DVD→Kit	70.87	72.95	73.70	74.20	74.43	75.03	**76.04**
Boo→DVD	75.78	78.39	78.54	80.40	81.75	82.20	**84.05**
Boo→Ele	71.18	73.21	73.93	74.77	76.05	77.06	**77.57**
Boo→Kit	73.43	75.61	76.07	78.12	76.68	77.36	**77.39**
Ele→DVD	72.35	75.77	75.81	77.02	78.35	80.02	**81.06**
Ele→Boo	70.87	73.36	73.30	75.05	76.17	77.97	**79.97**
Ele→Kit	82.91	85.48	85.55	87.35	84.78	86.15	**87.48**
Kit→DVD	73.98	76.96	76.55	78.70	78.94	79.41	**80.76**
Kit→Boo	82.03	83.78	85.08	85.29	85.85	86.78	**86.68**
Kit→Ele	73.63	75.50	76.07	77.35	78.09	79.66	**80.64**
Mean	74.20	76.58	77.05	78.26	78.75	79.79	**80.87**

*The best classification results are indicated in bold in Table.*

**TABLE 8:** F1-score results for each cross-domain task on English corpus.

Tasks	K-SVD	ARTL	DMTTL	SMITL	WAAR	SFA	TLDDL
DVD→Boo	74.08	76.90	77.49	78.03	80.13	80.85	**82.89**
DVD→Ele	70.08	72.18	73.25	73.96	74.70	75.72	**76.58**
DVD→Kit	71.54	73.13	73.66	75.07	74.81	75.71	**76.56**
Boo→DVD	76.05	79.53	78.79	80.89	82.42	82.84	**84.83**
Boo→Ele	71.25	73.94	74.43	75.42	76.43	77.37	**77.97**
Boo→Kit	73.69	75.84	76.58	78.37	77.18	77.68	**77.98**
Ele→DVD	72.62	75.97	75.92	77.24	79.11	80.51	**81.40**
Ele→Boo	71.16	73.39	73.77	75.17	76.50	78.57	**79.96**
Ele→Kit	83.05	85.39	86.34	87.63	84.83	86.56	**88.09**
Kit→DVD	74.77	77.37	76.77	78.98	79.37	80.09	**80.96**
Kit→Boo	81.86	84.19	85.30	86.36	86.36	87.12	**87.29**
Kit→Ele	74.06	76.26	76.83	77.50	79.01	79.94	**80.50**
Mean	74.52	77.01	77.43	78.72	79.24	80.25	**81.25**

*The best classification results are indicated in bold in Table.*

### Experiments With Different Training Samples in the Target Domain

**FIGURE 1:**
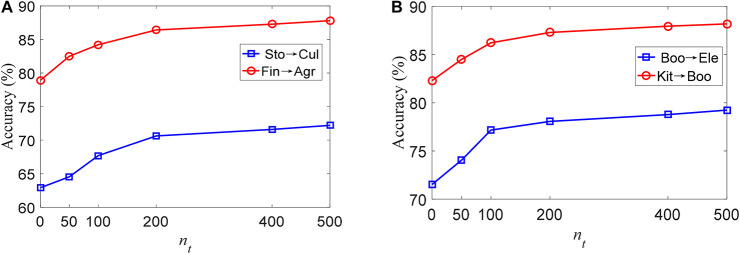
The accuracy of TLDDL with different size of ***n*_*t*_**, **(A)** Chinese corpus; **(B)** English corpus.

To show the influence of the number of training samples in the target domain on the proposed TLDDL algorithm, I compare its classification accuracy on the Chinese and English corpora in Figure 1. The size of *n*_*t*_ is set to be 0, 50, 100, 200, 400, and 500, respectively. Since the size of *n*_*t*_ should be less than that of *n*_*s*_, the maximum size of *n*_*t*_ is set to 500. The results of Sto→Cul and Fin→Agr on the Chinese corpus are shown in Figure 1A. When the size of *n*_*t*_ is 0, the classification accuracy of the two tasks is the lowest. With the increase of the size of *n*_*t*_, the classification accuracy is gradually improved. When the size of *n*_*t*_ exceeds 200, the classification accuracy reaches a relatively stable state. The results of Boo→Ele and Kit→Boo on the English corpus are shown in Figure 1B. I can see that the classification accuracy of TLDDL increases rapidly after adding the training samples in *n*_*t*_. When *n*_*t*_ reaches 200, the classification accuracy is stable and the growth rate of accuracy is small. Considering the practical application scenarios of transfer learning, 200 texts selected in the target domain for training are reasonable.

## Conclusion

In this study, I have developed a transfer learning classification algorithm for cross-domain text sentiment classification. Inspired by the advantage of dictionary learning in knowledge reconstruction and sparse representation, I proposed to employ subspace projection and transfer learning into the framework of dictionary learning. Considering the within-class minimizing and between-class maximizing of sparse coding coefficients, I define the following discrimination information preserved term in the objective function; meanwhile, I adopt the PCA term in the objective function to retain the discrimination knowledge. In such an algorithm, a domain-invariant dictionary is built to establish a connection between different domains. Experimental results indicate that the TLDDL algorithm achieves good classification performance. In the future, from the perspective of multiple learning strategies on dictionary learning, I will consider how to realize transfer learning of multi-source and heterogeneous data in the proposed algorithm. In addition, from the perspective of extracting features, I will investigate how to automatically implement feature selection and building a classifier in a model framework.

## Data Availability Statement

Publicly available datasets were analyzed in this study. This data can be found here: The toutiao-text dataset is addressed as: https://github.com/aceimnorstuvwxz/toutiao-multilevel-text-classfication-dataset. The Chinese Weibo dataset is addressed as: http://tcci.ccf.org.cn/conference/2014/pages/page04_sam.html. The Amazon review dataset is addressed as: http://jmcauley.ucsd.edu/data/amazon/.

## Author Contributions

SZ conceived and developed the theoretical framework of the manuscript, carried out experiment and data process, and drafted the manuscript.

## Conflict of Interest

The author declares that the research was conducted in the absence of any commercial or financial relationships that could be construed as a potential conflict of interest.

## Publisher’s Note

All claims expressed in this article are solely those of the authors and do not necessarily represent those of their affiliated organizations, or those of the publisher, the editors and the reviewers. Any product that may be evaluated in this article, or claim that may be made by its manufacturer, is not guaranteed or endorsed by the publisher.
